# How Do Molecular Systems Engineering Scientists Frame the Ethics of Their Research?

**DOI:** 10.1080/23294515.2024.2302994

**Published:** 2024-01-09

**Authors:** Renan Gonçalves Leonel da Silva, Alessandro Blasimme, Effy Vayena, Kelly E. Ormond

**Affiliations:** Health Ethics and Policy Lab, Department of Health Sciences and Technology, ETH Zurich, Zurich, Switzerland

**Keywords:** Molecular systems engineering, bioengineering, bioethics, qualitative methods, interview study, empirical research

## Abstract

**Background:**

There are intense discussions about the ethical and societal implications of biomedical engineering, but little data to suggest how scientists think about the ethics of their work. The aim of this study is to describe how scientists frame the ethics of their research, with a focus on the field of molecular systems engineering.

**Methods:**

Semi-structured qualitative interviews were conducted during 2021–2022, as part of a larger study. This analysis includes a broad question about how participants view ethics as related to their work, with follow up probes about the topics they consider most important. Interviews were transcribed, inductively coded by two researchers to consensus, and analyzed thematically.

**Results:**

Twenty-four scientists participated in the study. Interviewees hold positions as professors, principal investigators, and senior staff researchers in universities or research institutes in the United States and Europe. Among those scientists who reported reflecting on ethical considerations in their work, many equated ethics with research ethics topics (e.g., safety, replicability), or with regulation and guidelines. Participants expressed the view that ethical issues are primarily relevant for clinical trials of bioengineered products, or for those working with animal or human subjects. Scientists described their research as “too early” or “not examining anything living” with regard to ethical reflection. Finally, many felt that ethics is seen as territory for experts and therefore beyond scientists’ competencies.

**Conclusions:**

Molecular systems engineering scientists currently focus on regulatory aspects as the framework for their ethical analyses. They describe using a framework to define when life arises, as a means to determine when further ethical engagement is warranted. Further research is needed to investigate how scientists relate to the ethics of their scientific work, and build consensus around concepts of life, autonomous behavior, and physiological relevance of bioengineered systems.

## Background

In recent years, scientists, engineers, and other stakeholders have developed new capabilities to precisely engineer complex responsive materials, molecular systems, and life-like synthetic cells. This emerging field of bioengineering called “molecular systems engineering” (MSE) can be described as an interdisciplinary area of research promoting “the convergence of molecular-scale engineering and a systems biology approach that looks at how individual pieces of a biological organism fit together in an integrated fashion, and how such system-scale interaction can be modelled and quantified” (da Silva and Blasimme 2024). Engineered molecular systems not only mimic biology, but can also improve it, using technology to expand organisms’ functional abilities, enabling them to cope with deficits caused by injury or disease.

Since MSE investigates the possibility of technological systems that integrate living and artificial parts, it is not surprising that there is discussion about the ethical implications (Akin et al. 2017; Bauer and Bogner 2020). Concerns include whether natural scientists and engineers can guarantee transparency and accountability in data reproducibility, precision design and engineering techniques, manufacturing standards, responsible design, and risk assessment of synthetic bioengineered systems and tools (Balmer et al. 2015; Sun et al. 2022; Liao et al. 2023). This ongoing discussion (da Silva and Blasimme 2024; Heidari, Elger, and Stutzki 2016) led us to think about the ethical and societal implications of such research, including whether and how ethical considerations are framed by scientists in their daily work. We are not aware of any empirical research exploring the values, norms, and ethical beliefs informing the activities of MSE scientists. Therefore, the aim of this study is to investigate how molecular systems engineers frame the ethics of their research

## Methods

We conducted exploratory semi-structured interviews with active researchers in the field of MSE. The research protocol received approval from the Ethics Commission of ETH Zurich (2021-N-71).

### Subjects and recruitment

Researchers holding leading roles as directors or scientific coordinators of national scientific consortia in Europe and United States were prioritized, as well as university faculty members, senior staff scientists, and postdocs conducting experimental and applied research in MSE-related technologies. Interview invitations were guided by a preliminary search for publications in the field of MSE using the software Dimensions.ai. (Digital Sciences & Research Solutions, access granted by the Department of Health Sciences and Technology of ETH Zurich). We also used a “snowball” recruitment approach, obtaining recommendations for additional participants from interviewees, selecting for gender balance, variety in institutional affiliation, and intensive work in research and development of engineered molecular systems and technology. Sixty-five invitations were sent *via* e-mail between May 2021 and July 2022.

### Interviews

Interviews were done as part of a more extensive research plan collecting data about how scientists define “molecular systems engineering” and initiatives adopted by science teams to organize the production of knowledge in this emerging field of research. An interview outline was developed by the first author (RS, a Science Technology and Society researcher) in consultation with the coauthors, exploring processes that scientists adopt to address ethical questions that arise during their research, the role that scientific discipline might play, and the familiarity of scientists with stakeholders engaged with ethical considerations. It aimed to collect evidence about three dimensions of researcher activities: (1) How researchers frame the disciplinary configuration, shared rationales, and consensus around scientific practices in the laboratory, and the scope and aims of their work, (2) Ethical and regulatory issues that researchers face, and (3) How researchers perceive bottlenecks in science policies, and in communication and the future of their field. Six specific ethics-related questions from the interview that pertain to this sub-study are listed in Table 1.

**Table 1.** Interview questions.

Ethics-focused questions	How do you manage eventual questions or discussions about ethical problems related to your research projects coming from your science team? Do you anticipate any ethical issues/complications that people working in your research projects could face? Could you give me one example (e.g., unintended mutations of CRISPR-Cas-9 genome editing)? What are the main ethically controversial situations you’ve been exposed to in you work in this field? Are those issues specific to MSE, or common to other areas such as synthetic biology, genome editing, regenerative medicine etc.? How did you overcome those ethical issues collectively (e.g., following ethical guidelines, ethics committee, etc.)

Other topics emerged as important drivers of scientific practice in MSE and will be illustrated in separate publications, including how concepts of MSE circulate through scientists’ field definitions; organizational problems related to clinical translation of bio-architected tissues; and questions related to the limits of science communication, science policies, public engagement, and trust in their field.

Interviews were conducted by a single interviewer (RdS), with most taking place remotely (*n* = 17) *via* Zoom between June 2021 and August 2022. The remaining interviews (*n* = 7) were conducted in person over the course of a month of fieldwork in August 2022 at the Brookhaven National Laboratory in the United States, in order to collect broader empirical evidence about ethical issues faced by MSE researchers in a specific organizational setting.

Field notes were produced during online and in-person interactions. Interviews lasted an average of 45 min, for a total of 18 h. The first author determined the endpoint based on data saturation, after completion of the ethnographic work. Participants did not receive transcripts, in order to preserve the contextual analysis of narratives, in line with standards for semi-structured interviews in social science research (Knott et al. 2022). Editing could result in the loss of important details from the researcher’s activities in scientific settings, for example in chance interactions and spontaneous answers.

### Data analysis

Interviews were recorded, and transcribed by a professional anthropologist (*n* = 16) and using the AI Software TRINT (*n* = 8). Transcripts were checked for accuracy and data familiarization, and then de-identified. A codebook, focused on how scientists think about ethics in their field, was co-designed inductively by two researchers (RdS and KO). These two individuals conducted pilot coding to consensus on two rounds of transcripts, and addressed discrepancies to increase accuracy and refined the codebook to improve its precision and clarity (Abbadia 2023). At that point, all transcripts were coded to consensus by the same two researchers and thematically analyzed (Braun and Clarke 2006) with the assistance of Atlas.ti. While there were 6 specific ethics-focused questions in the interview guide, entire transcripts were coded using the ethics-focused codebook, since it was clear from codebook development that relevant points arose in response to broader questions.

## Results

### Study participants

In total, 24 MSE scientists participated in the study. Participant characteristics are reviewed in Table 2. Interviewees hold positions as professors, principal investigators, staff senior researchers, and referenced post-docs working in the area of MSE. Thirty-three percent of interviewees self-identified as female, and 67% hold positions as professors and directors of research institutes in universities or institutes of technology in the United States, Switzerland, Germany, Netherlands, and the United Kingdom.

**Table 2.** Study participant characteristics (*n* = 24).

Participant characteristics	n	%
Sex		
Male	16	67
Female	8	33
Career level (equivalent)		
Professor	10	42
Associate Professor	1	4
Assistant Professor	3	13
Staff Scientist	5	21
Project coordinator/manager	3	13
Postdoctoral researcher	2	8
Institutional affiliation		
Universities and research institutes	16	67
National Laboratory	7	29
R&D department—Industry	1	4
Disciplinary domains		
Science, Technology, Engineering and Mathematics	17	71
Biological and Biomedical Sciences	6	25
Health Sciences	1	4
Country		
United States	10	42
Switzerland	8	33
Germany	3	13
Netherlands	2	8
United Kingdom	1	4
Work roles		
Research and teaching	12	50
Research only	9	38
Management only	3	13

#### Main themes

Our thematic analysis grouped findings under three themes: “Organizational culture” focused on how scientific rules and institutions influence scientists’ behavior toward ethics; “Defining Ethics” showing how they described their own ethical reflection both by equating it to research ethics, regulation or as something peripheral in their research, and “Ethics as only within the realm of experts.”

### Organizational culture

In our study, respondents often viewed ethical awareness as dependent on the interests of individual scientists or the culture of a team or organization.

It will depend very much on the personality. (…) There are some personalities that do think about ethical issues or societal issues of the impact of what they’re doing. I would say these are the minority. Nanomedicine Professor.

So they [MSE scientists] do not find an environment that is inclined to discuss this kind of issue unless there’s something formal or organized by the head of the lab, or if we go to an event, or if we organize something specific around ethics… But that is not a routine kind of daily exercise. Biological engineer.

Participants who work in more controversial areas of research cited examples of circumstances in which they consider ethics.

One concern that we had with the biohybrid robotics was that currently the best beating cells are fresh cardiomyocyte from rat babies, and my student refused doing this because driving a robot from just killing rat babies was maybe ethically not super great [participant implied “unethical”]. So we changed the cell system. I think this is something that [ethics] very clearly had an impact. Biophysics Professor.

### Defining ethics

#### Equating ethics with regulation

Study participants discussed their ethical considerations by primarily describing rules and criteria that promote safety, accuracy, replicability, and reproducibility, and the regulatory requirements of their projects.

For example, many scientists described their knowledge of regulations as applied to their research.

I think overall they have an elaborate process that maintains, you know, appropriate ethics but also appropriate safety regulations are in place. … the most important thing is that all safety procedures are also followed. Synthetic biology Professor.

The regulation is quite strict of course, because it’s an advanced therapy product. So it’s the highest level of risk. We manipulate cells and transplant it into patients. (…) so the regulation is quite strict. It creates a lot of work [that] is necessary to protect patients. Clinician Scientist.

Similarly, some described a focus on research conduct as an ethical issue, as it improves scientific rigor and enables reproducibility of findings.

there are a lot of research conduct issues in terms of making sure that anything that we publish is rigorous and reproducible, and that we’re being very transparent with the things that we do. You know, we do think about that and we value that very highly. (…) If there’s something that we’re not confident that we can reproduce (…) like we need to put the pause on this until we go bask and we figure out, either why it is that we can’t reproduce it or start to reproduce it again. Those are the kinds of questions that we discuss and that we talk about. Professor of Chemistry.

Awareness of ethics, vis-a-vis regulatory paperwork or bureaucratic procedures required for projects, was presented as primarily relevant for MSE research involving animal or human subjects.

There’s a lot of ethical considerations related to animal research that we do. But [because] we do a lot of work with mice and mice models, there’s a very detailed ethical procedure in the mouse protocol (…). Biotechnology Professor.

Finally, interviewees described following the rules of local ethics committees, professional guidelines (e.g., International Society for Stem Cell Research ISSCR), and protocols and regulatory rules (e.g., Federal Office of Public Health) when applicable for research with embryonic stem cells.

We use the guideline from the International Society for Stem Cell Research. They bring out guidelines for you to know how [to] work with human stem cells. They just updated it recently for gastroloids, embryoids, etc. (…) Locally, we request approval of ethics committees to work with the human stem cells. (…) we also have to go though [named the committee] for embryonic stem cells. Developmental biology Professor.

#### Ethical concern as premature

Study participants gave examples of circumstances in which ethical thinking is relevant, or premature. When asked “do you think about the ethical issues of your current project?,” many participants voiced a belief that ethical considerations are relevant only for projects involving animal research or clinical trials.

I mean the closest we’ve gotten to like human material or animal studies at this point has been we’re using some human cell lines. (…) We certainly don’t think about animal studies very carefully at the moment, because we haven’t gone down that path just yet. Professor of Chemistry.

So far, not really. Because we’re not at the point where anything is really transferred, you know. Molecular biology researcher.

Similarly, a researcher pointed the irrelevance of thinking about the ethics of their work due to not being working with technologies related to genomic editing or genetic manipulation

I haven’t got to the point in my research to discuss about the ethical part of it, but I’m not doing anything that will perturb like… for example, CRISPR/Cas[9] gene editing that will have ethical impact. Postdoctoral researcher in biomedical engineering.

Some scientists took a more nuanced approach, describing their technologies as far being from clinical application, but agreeing that eventually ethical considerations will be relevant in assessing a technology’s cost-benefit ratio.

I don’t know. I’m very far from those types of application [referring to a conversation that came up spontaneously about adverse reactions nanomedicines] (…) But I could imagine, you know, that not everything which will be developed using molecular engineering will be absolutely harmless, but you are seeing cost benefit analysis. (…) I think science and what we create in science, it doesn’t have this label being bad or being good. Physics Professor.

The understanding of what counts as a living organism appears critical to how respondents distinguish between projects that warrant ethical scrutiny and those that don’t. Some researchers expressed a selective view about which organisms should be subject to ethical scrutiny, often dismissing potential ethical issues when working with “small” organisms like yeast or bacteria.

We honestly don’t think that much about this particular questions. I mean we’re working with laboratory strains of mostly yeasts and they are not very good at surviving [laughs]. We’re not really that concerned that they’ll get out and do something weird. Chemical engineering professor.

I am in the fortunate situation that nothing that we do actually raises any ethical concerns. We don’t fiddle with the DNA of living systems other than bacteria. One could argue why. But nobody care[s] about whether a bacteria is happy or not. It makes life quite easy for us. Professor of chemistry.

Many interviewees have views about what a living organism is, and which living organisms matter from an ethical standpoint. Participants often reflected that they do not think more about ethics because they work with molecular systems or life-like artifacts, which they consider far from becoming life. And because they do not believe that they are dealing with “living organisms,” they maintain MSE is “more [about] safety than ethics.”

We’re not working with living organisms, right? We only have to smash them apart to get the building blocks, but we’re not creating new organisms. So that’s why in terms of ethics there’s not much involved. Secondly, we’re never working with human material. It’s all microbiology type of material, so… So it’s more [about] safety rather than ethics I would say. Professor of Chemistry.

Views are different—and more cautious—when scientists discuss synthetic cell research. Still, some participants felt that ethical considerations about synthetic cells should not be a matter of concern, since they remain far from behaving as living cells.

We’re still far away from anything that looks like a living cell if you want to approach it from that side. I’m not too worried about that the public opinion will go against us. Professor of synthetic biology.

However, synthetic cells may become a matter of concern due to the way press speak about them.

To some extent of course there is nothing unethical until you have a synthetic cell. So in that respect we are very far away from doing anything unethical. (…) So eh I think we have spent more time on looking at people’s perception on synthetic cells. And of course it’s easy to talk about Frankenstein cells and stuff like that. (…) You’re not trying to build something scary. Professor of chemistry.

#### When ethical concern might be warranted

For many participants, ethical scrutiny is warranted for cases in which experimental activates can be seen as creating new forms of life.

Ethical aspects are also not issues to us. What can be a challenge, which is also seen as an ethical question (…) is the potential creation of life. [In our project] we had few but very strong objections, including from a very influent scientist in synthetic biology, against the idea to build fully functional, or “living,” synthetic cells. I don’t know what the reason was actually, but I think these reasons are either religious motivations or maybe a philosophical insight. Synthetic Biology researcher.

Interviewees felt that ethical consideration is also needed if technologies like complex biomimetic systems develop a sufficient level of autonomy or physiological relevance to be considered a living thing.

As soon as we make something synthetic that starts to live or have an own autonomous reaction you have to think about that of course. When you engineer cells and their functions that’s absolutely clear. Biophysics Professor.

Even in the case of synthetic cells (regarded by synthetic biologists as a frontier where living systems could potentially emerge) and organoids, our study participants considered synthetic cell research in potentially controversial areas such as brain organoid research to be incipient, and ethical concern or fears thus premature.

… I have been thinking about the ethical issues that could arise (…). There are some papers on brain organoids discussing what the ethical issues could be. But when we start the project, I mean, this is not in my mind day-to-day because I know the huge limitations of these systems. This is a bunch of pregenital cells that start to acquire fades. (…) it’s not that we have really a human brain in our hand. Developmental biology Professor CT01.

Brain organoids in particular are deemed far from developing features that could foreshadow human-like characteristics.

If we think about the different tissues we have of course the brain organoids would be the ones that are closest to raise any issues (…) [But] these brain organoids don’t have any consciousness and they are really early developmental systems. (…) if we say, ok let’s add a vasculature, immune cells, let’s make it the best possible brain tissue, and let’s grow it for one year. If it turns into a real physiologically relevant system even after one year then one has to start eh… one runs into ethical concerns.

Finally, some researchers explained that reviewers and funding agencies do not demand scientists go too deep into the analysis of ethical problems associated with their research.

I think maybe it’s too early to discuss that. [It would be the case] only if this field really becomes big and starts to push [named an agency] and other funders to have the attention from reviewers for this problem. But it’s really too early for that. Biophysics Professor.

### Ethics as only within the realm of experts

Many respondents see ethics as a domain that goes beyond scientists’ competencies and skills, and should thus be delegated to experts in that field.

Ethics in the way in which they practice science? There are different angles of ethics. (…) I think there’s no lack but maybe more distancing from ethical concerns… Because engineers, chemists, or technology developers, do not consider themselves as experts. Ethics is not their territory. Nanomedicine Professor.

Some participants explained that their projects engage regulatory experts to navigate any required steps, positioning themselves as only indirectly involved with the ethical considerations of their research.

We have a full team which is working on this [regulation]. These are huge packs of documentations which are not simple to decipher and to interpret. So it requires experts. (…) it’s it’s a field of its own. And it is extremely important to have this expertise imbedded because we cannot trust that a scientist will just read an article and understand what is necessary. Tissue engineering Professor.

Others cited experts in the field of ethics, for example an international congress that involved bioethicists.

Usually [bioethicists] meet with us on this annual meeting of [named the conference]. And there’s a lot of discussion. They always have ethics sub-symposiums and so on. Tissue engineering researcher.

## Discussion

Our study presents a sampling of how MSE scientists frame the ethical dimension of their research. When they do consider ethical issues, scientists primarily focus on research ethics issues such as safety, accuracy, and reproducibility of research findings, as well as regulatory considerations. Their ethical focus is framed around the belief that their research does not involve living organisms holding ethical relevance. Many researchers emphasized that they work with bioengineered artifacts that are too simple or rudimentary to exhibit ethically relevant features, such as being considered living organisms, or faithful replicas of human anatomical parts or functions. And because ethics as a broader topic is seen as an experts’ domain, ethical analysis is considered relevant mostly with respect to clinical trials or animal experimentation.

This study demonstrates that at least some MSE scientists restrict their ethical thinking compared to how bioethicists think about ethics, and it indicates several reasons why scientists may do so. This interpretation relies on potential differences in the way we (the authors, as bioethicists) and participants (as MSE scientists) understand what ethics is. While both scientists and bioethicists are concerned with ethics, our approaches, roles, and areas of expertise differ significantly. Our interviewees primarily focus on addressing ethical issues as they arise (for example, when submitting for ethics committee review or following regulations about their research), while bioethicists specialize in the systematic study of ethical issues in the context of biology, medicine, and healthcare, providing guidance and analysis from a broader ethical perspective, including forecasting potential ethical challenges in the future.

Our findings mirror Wolpe (2006) and McCormick et al. (2012), who identify a significant lack of awareness about ethics amidst life scientists (e.g., considerations about implications of manipulating genetic data, creating synthetic living/autonomous organisms, biological risk of new biotechnologies, or potential misuse of new bioengineered systems). These studies also identified the sentiment that “scientific work has little to do with ethics” (Wolpe 2006, 1023), and that scientists “are unaware that their work does in fact have ethical and societal implications” (McCormick et al. 2012, 44). Evidence about reasons for the perceived lack of relevance of ethics for scientific practice is not unique to MSE. In search of potential causes, Ziman argues that the behaviors of individual scientists are affected by the way they are trained to be part of successful scientific teams; this helps to explain why questions of ethics (and most anything not directly related to the mission of advancing scientific knowledge) tend to be sidelined (Ziman 1998). According to the literature, thinking systematically about ethics can be seen as an obstacle to scientific progress (Pickering 1992; Pickersgill 2012; Jach 2019), noise that could compromise the objectivity and impartiality of science (Ziman 2000, 2001), a task beyond their research duties (Kennedy 1997), or a subject to be addressed at the individual level (Rutjens and Heine 2016).

The notion of “perceived proximity” proposed by Ladd et al. (2009) can also be helpful for understanding how scientific systems (and the individuals within them) respond instrumentally to social awareness of ethical concerns. According to Ladd and colleagues, perceived proximity is a collective perception shared among scientists about how sensitive they are in regards with societal awareness about what they do in their labs” (Ladd et al. 2009). When controversies and fears become public, the perceived proximity between scientists and society narrows, increasing scientists’ need to obtain societal acceptance for their work (Bauer and Bogner 2020), therefore increasing the importance of ethical considerations. For example, some scientists in our study identified fear of potential unintended consequences of engineered molecular systems for humans, as elements that prompt ethical controversy. Additionally, when a respondent said “nobody cares about whether a bacteria is happy or not”; the building of societal consensus about which organisms are relevant for ethical scrutiny demonstrates perceived proximity through the adoption of a collective “selective conduct” about what constitutes living organisms, and even which living organisms are important to care about. As a result, scientists’ awareness about ethics is often activated to gain trust and societal acceptance, including to convince regulators and other experts that they are working responsibly (Torres-Padilla et al. 2020). As argued by Wolpe (2006), these approaches typically invite scientists to adopt an instrumental approach to ethics, framing discussions about the ethics of science regarding the risks and safety of scientific research (Wolpe 2006; Fujimura 2013; Hesselmann and Reinhart 2019). While relevant, such attitudes might fail to recognize the complexity of the ethical issues that emerge in cutting-edge scientific research.

Institutions also impact how scientists think about ethics. For example, the notion of “scientistic worldview” (Jach 2019) i.e., “(…) a form of worldview characterized by the tendency to justify one’s beliefs and behavior with scientific findings and to function based on the theorems formulated by scientists" (p. 2) can be instrumentally applied to analyze how scientists frame ethical questions, and in our study relates to the tendency to reserve ethics for dedicated experts. In the research literature, scientists are reported to leave ethics to the experts in order to allocate their own time more efficiently (Wolpe 2006) or to promote a sense of transparency by subjecting their work to broader social scrutiny (Novossiolova and Sture 2012); our participants did not mention these perspectives. In contrast, several participants described some elements of organizations (such as ethical review committees and granting agencies) as institutionalizing late appreciation of ethics in science, which has also been noted in the literature (Hunckler and Levine 2022).

Individual values and beliefs are also important for understanding how scientists frame ethics (Greene et al. 2009). In our study, discussions about the moral relevance of research in MSE are closely related to how individuals understand the concept of life, and to their beliefs about the ontological and moral status of the systems they work with or create. Their ethical thinking is framed around the belief that their research does not involve living organisms holding ethical relevance in society. According to study participants, the manipulation of biomolecules, synthetic cell-like systems, or multi-cellular/bioengineered tissue constructs are not yet the subjects of ethical scrutiny because they do not involve living organisms, or their molecules and functional compartments are “too small to care.” Many researchers emphasized that they work with bioengineered artifacts that are too simple or rudimentary to exhibit ethically relevant features, such as being considered living organisms, or faithful replicas of human anatomical parts or functions. Participants also gave many examples of why synthetic DNA or a bacteria were not perceived as creating ethical issues, while animal experimentation with biohybrid robots were. This discussion reflects the idea that systems like “organoids are more than cells but less than organisms” (Hinterberger and Bea 2023, 5). Categorical distinctions between living and non-living entities, naturally occurring organisms and laboratory models, natural kinds and artificial ones are ubiquitous in conceptual (ontological) discussions about the life sciences. Present-day discussions about the nature of stem cell-derived human embryo models and their moral and legal status clearly show the complexity of such demarcations (Blasimme and Sugarman 2023).

Several proposals can be put forward in response to our findings. First, we believe that educational processes need to extend beyond the traditional Regulatory and Responsible Conduct of Research (RCR) focused approach. Instead, scientists should be equipped with the skills and knowledge to "think about ethics" from the very outset of their research endeavors. This entails a shift in how ethics are defined, moving away from a compliance-based perspective and moving toward a more comprehensive understanding of ethical considerations of how technology impacts individuals and society in both good and harmful ways. As an example, science education programs could incorporate modules that understand historical examples of unethical research outcomes, and to encourage critical ethical reflection, helping researchers develop a nuanced ethical framework that guides their work at all stages of their projects, from conception to completion.

Secondly, we feel that it is critical to addressing the perception that ethics experts and scientists work in isolation. To bridge this gap, institutions and funders should actively promote interdisciplinary collaboration throughout the research continuum. Ethics experts, along with scholars from humanities and social sciences, should be embedded into the early stages of research projects, including project conception, grant writing and consortium formation. An ongoing integration of ethics specialists into the project management process could produce multiple benefits. It can contribute to the transformation of organizational culture within research institutions by fostering a culture of ethical awareness and responsibility. Moreover, it can enhance individual awareness among scientists about the ethical dimensions of their work. For example, researchers in fields like genetics could collaborate with ethicists to address complex questions about the ethics of gene editing, biobanking or genomic datasharing practices, or the history of eugenics and its relationship to health equity in genetic testing and treatments in today’s world.

Finally, it will take more comprehensive science and technology policies to foster those types of collaboration. It involves making the ethical thought process more inclusive and participatory. For instance, involving patient advocacy groups, community representatives, and other nonscientific voices in ethical discussions can lead to more context-sensitive ethical guidelines (Beier, Schweda, and Schicktanz 2019). This approach not only ensures that ethical decisions are more representative but also helps build trust between the scientific community and society.

This study has limitations. First, there is a potential bias in the disciplinary configuration of our sample, since MSE research is mainly composed of scientists from chemistry, physics, and materials sciences. In addition, the inclusion of scientists exclusively from academic institutions in high income countries means that this study presents a restricted picture of the diversity of initiatives and attitudes toward ethics in this field internationally—a similar study in countries like China, South Korea, or high income countries as Japan, where robust science is being developed in this field, would certainly broaden the findings.

## Conclusion

Our study, then, brings new evidence about how scientists working in MSE make sense of the moral and philosophical issues that result from their work in manufacturing and experimentation with things that do not exist in nature. Further research is needed to investigate how MSE scientists negotiate and build consensus around shared concepts of life, autonomous behavior, and physiological relevance, through the implementation of new institutionalized forms of governance of knowledge, risk, and accuracy of their science and technology. In the context of empirical bioethics research and science studies, these ideas hold immense importance for shaping how science is socially constructed among them.

The insights gained from this study can assist bioethicists and scholars from diverse fields in advancing a more nuanced and interdisciplinary comprehension of what holds moral significance to scientists in their day-to-day activities within laboratories. It also sheds light on how scientists collaboratively shape their perspectives and cultivate shared notions concerning life, risk, and regulation, which in turn impact their institutions and their frameworks for producing expert knowledge in society. By reimagining how ethics are integrated into scientific research by emphasizing early education, fostering interdisciplinary collaboration, and more ethically-awareness-driven science and technology policies. Implementing these proposals, scientific research can ultimately contribute to the development of sciences that does not pretend to be only technically sound but also socially embraced.
